# Development of *Salvia officinalis*–Based Self-Emulsifying Systems for Dermal Application: Antioxidant, Anti-Inflammatory, and Skin Penetration Enhancement

**DOI:** 10.3390/pharmaceutics17020140

**Published:** 2025-01-21

**Authors:** Krisztina Bodnár, Boglárka Papp, Dávid Sinka, Pálma Fehér, Zoltán Ujhelyi, István Lekli, Richárd Kajtár, Fruzsina Nacsa, Ildikó Bácskay, Liza Józsa

**Affiliations:** 1 Department of Pharmaceutical Technology, Faculty of Pharmacy, University of Debrecen, 4032 Debrecen, Hungary; bodnar.krisztina@pharm.unideb.hu (K.B.); papp.boglarka@pharm.unideb.hu (B.P.); sinka.david@pharm.unideb.hu (D.S.); feher.palma@pharm.unideb.hu (P.F.); ujhelyi.zoltan@pharm.unideb.hu (Z.U.); bacskay.ildiko@pharm.unideb.hu (I.B.); 2 Doctoral School of Pharmaceutical Sciences, University of Debrecen, 4032 Debrecen, Hungary; lekli.istvan@pharm.unideb.hu (I.L.); kajtar.richard@euipar.unideb.hu (R.K.); 3 Department of Pharmacology, Faculty of Pharmacy, University of Debrecen, 4032 Debrecen, Hungary; 4 MEDITOP Pharmaceutical Ltd., Pilisborosjeno Ady Endre Street 1, 2097 Pilisborosjeno, Hungary; fruzsina.nacsa@meditop.hu

**Keywords:** *Salvia officinalis*, dermal drug delivery, SNEDDS, anti-inflammatory effect, antioxidant effect

## Abstract

Background/Objectives: The present study focused on the formulation and evaluation of novel topical systems containing *Salvia officinalis* (sage), emphasizing their antioxidant and anti-inflammatory properties. Sage, rich in carnosol, offers considerable therapeutic potential, yet its low water solubility limits its effectiveness in traditional formulations. The aim of our experimental work was to improve the solubility and thus bioavailability of the active ingredient by developing self-nano/microemulsifying drug delivery systems (SN/MEDDSs) with the help of Labrasol and Labrafil M as the nonionic surfactants, Transcutol HP as the co-surfactant, and isopropyl myristate as the oily phase. Methods: The formulations were characterized for droplet size, zeta potential, polydispersity index (PDI), encapsulation efficacy, and stability. The composition exhibiting the most favorable characteristics, with particle sizes falling within the nanoscale range, was incorporated into a cream and a gel, which were compared for their textural properties, carnosol penetration, biocompatibility and efficacy. Results: Release studies conducted using Franz diffusion cells demonstrated that the SNEDDS-based cream achieved up to 80% carnosol release, outperforming gels. The 2,2-diphenyl-1-picrylhydrazyl (DPPH) test and enzyme-linked immunosorbent assays (ELISA) showed strong efficacy, with an in vivo carrageenan-induced rat paw edema model revealing that the SNEDDS-based cream significantly reduced inflammation. Conclusions: These findings highlight the potential of SNEDDS-enhanced topical formulations in improving therapeutic outcomes. Further research is warranted to confirm their long-term safety and efficacy.

## 1. Introduction

Our skin, as the largest organ of our body, plays a key role in maintaining our health and acts as a barrier against mechanical and physical damage, pathogens, and chemicals. Healthy skin is elastic, hydrated, and evenly colored. However, skin diseases significantly impair our quality of life and can even lead to serious health problems [1]. Topical drug delivery systems (TDDSs) have garnered significant attention in recent years due to their ability to deliver active compounds directly to the site of action, particularly in the treatment of inflammatory skin conditions such as dermatitis and psoriasis [2]. Among various topical formulations, creams and gels are commonly used due to their versatility and ease of application. However, the successful delivery of poorly water-soluble compounds remains a key challenge in the development of effective TDDSs. Natural active ingredients, such as those derived from plants, are increasingly being incorporated into these formulations for their anti-inflammatory, antioxidant, and antimicrobial properties [3]. Traditional medicinal plants provide interesting and largely unexplored resources for the development of new potential cosmetic and pharmaceutical products [4].

The development and application of plant-derived active compounds, such as polyphenols, terpenoids, and alkaloids, have demonstrated significant potential in therapeutic formulations due to their diverse biological activities, including anticancer properties. However, their clinical translation is often hindered by substantial pharmacokinetic limitations, such as low bioavailability, rapid metabolism, and insufficient stability. Recent studies underscore the necessity of advanced delivery systems to overcome these challenges and enhance the therapeutic efficacy of these compounds. For instance, incorporating phytochemicals into nanocarriers, such as liposomes, nanoparticles, and biomimetic vesicles, has been shown to improve bioavailability and targeted delivery, addressing key pharmacokinetic barriers. Furthermore, strategies such as embedding plant-derived actives into cell-derived vesicles or hybrid bioinspired systems have demonstrated potential to enhance solubility and specificity while reducing toxicity and immunogenicity. These approaches not only improve drug pharmacokinetics but also present promising directions for advancing the therapeutic outcomes of phytochemical-based formulations [5,6]. Sage (*Salvia officinalis*, *S. officinalis*) has been a revered medicinal plant for millennia, with its healing properties recognized by ancient civilizations. Its constituents have been widely used for their anticancer, anti-inflammatory, analgesic, antioxidant, antimicrobial, and antimutagenic properties [7,8,9,10,11].

The antioxidant capacity of *S. officinalis* is attributed to polyphenols, flavonoids, diterpenes, and rosmarinic acid, which help neutralize free radicals and reduce oxidative stress in the body [12,13]. One such natural compound is carnosol, a polyphenolic diterpene found in the leaves of the sage, known for its potent anti-inflammatory and antioxidant activities [14]. According to the literature, carnosol exhibits radical scavenging activity comparable to α-tocopherol (vitamin E) [15,16]. It is formed from carnosic acid during the oxidation process that occurs during plant harvesting and drying under oxygen exposure [4]. Both carnosic acid and carnosol are abundant in the leaves of these plants, with their concentrations increasing as the plant ages [17]. Dried sage leaves can contain between 0.1% and 7% carnosol, depending on the variety and growing conditions [18,19]. Carnosol has also demonstrated the ability to modulate inflammatory markers and cytokines in various models, suggesting its potential for managing inflammation [20,21]. Previous studies have primarily focused on the in vitro and in vivo biological activities of carnosol, such as its ability to inhibit tumor growth by modulating key signaling pathways, including nuclear factor kappa B (NF-κB) and signal transducer and activator of transcription 3 (STAT3) [22]. For example, research has shown that carnosol exhibits potent anticancer effects against breast, prostate, and colon cancers by inducing apoptosis and reducing oxidative stress. However, many of these studies were conducted using simple formulations, limiting their translational potential due to challenges such as poor solubility, low stability under physiological condition, and rapid metabolism [23,24]. These characteristics result in low bioavailability, making it challenging to achieve effective therapeutic concentrations. Its pharmacokinetic profile suggests a short half-life and limited absorption when administered in conventional formulations [25]. In contrast, the present study aims to overcome these limitations by formulating a novel drug delivery system containing *S. officinalis* extract to enhance the solubility, stability, and permeability of the active ingredient. This approach not only improves the pharmacokinetic profile of carnosol but also provides a basis for its potential clinical application. Compared to previous studies, our work emphasizes the development of a delivery system that ensures consistent bioavailability while maintaining therapeutic efficacy.

As aforementioned, the clinical application of carnosol is hindered by its poor water solubility and limited bioavailability, which restricts its effectiveness when used in topical formulations [26,27]. Therefore, enhancing its solubility and penetration through the skin is crucial for maximizing its therapeutic potential. To achieve better absorption, self-emulsifying drug delivery systems (SEDSSs) were developed. These systems, comprising lipids, surfactants, and co-solvents, enhance the bioavailability of drugs, particularly lipophilic molecules such as carnosol. When administered orally or topically, the system spontaneously forms an emulsion when in contact with body fluids, thus facilitating faster and more efficient drug absorption [28]. Self-microemulsifying drug delivery systems (SMEDDSs) are a refined version of SEDDSs that produce microemulsions with droplet sizes in the range of 100 to 250 nanometers. The smaller droplet size increases the surface area for drug absorption and enhances stability. SMEDDS formulations generally provide improved bioavailability compared to SEDDSs and are particularly effective for drugs with low solubility and permeability [29]. Self-nanoemulsifying drug delivery systems (SNEDDSs) are characterized by their ability to form nanoemulsions with droplet sizes typically ranging from 1 to 100 nanometers. The reduced droplet size results in an even greater surface area and improved drug solubilization, absorption, and stability. SNEDDSs are particularly suited for delivering drugs with very low water solubility, offering enhanced bioavailability and the potential for targeted delivery [30,31].

SNEDDSs are usually defined by key characteristics, including particle size, zeta potential, droplet morphology, emulsification efficiency, polydispersity, and stability. Despite its benefits, it is important to note that SNEDDSs also present challenges, including stability, compatibility, and toxicity issues. Nonetheless, SNEDDSs hold significant potential in pharmaceutical applications, particularly in targeted drug delivery [31]. Nanosized drug carriers, such as nanoemulsions, facilitate the transport of drugs through the stratum corneum by creating a reservoir effect or disrupting lipid bilayers, thereby improving drug delivery to deeper skin layers [32].

The aim of this study was to formulate creams and gels incorporating the active ingredient into a self-nanoemulsifying drug delivery system (SNEDDS) to enhance the bioavailability of *S. officinalis*. The Franz diffusion method was used to model the release of carnosol from the carrier. To ensure the safety of the developed products and their ingredients, a 2-(4,5-dimethyl-2-thiazolyl)-3,5-diphenyl-2H-tetrazolium bromide (MTT) viability test was conducted on human keratinocyte (HaCaT) cells. An enzyme-linked immunosorbent assay (ELISA) was used to examine the anti-inflammatory effect. In vitro antioxidant capacity tests (2,2-diphenyl-1-picrylhydrazyl (DPPH)) were performed to assess direct radical scavenging activity. The in vivo anti-inflammatory effect of the chosen preparations was investigated by measuring rat paw edema.

## 2. Materials and Methods

### 2.1. Materials

*Salvia officinalis* leaf extract was obtained from Herbal Discont (Medinvest Hungary Ltd., Budapest, Hungary). Labrasol, Labrafil M 1944 CS, Cremophor RH 40, and Transcutol HP were procured from Gattefossé (Lyon, France), while Pemulen TR-1 and Carbopol 974P were purchased from Lubrizol Corporation (Wickliffe, OH, USA). Stearic acid, cetylstearyl alcohol, propylene glycol, isopropyl myristate, triethanolamine, and glycerol were sourced from Hungaropharma Ltd. (Budapest, Hungary).

The MTT [2-(4,5-dimethyl-2-thiazolyl)-3,5-diphenyl-2H-tetrazolium bromide)] dye, Dulbecco’s Modified Eagle’s Medium (DMEM), phosphate buffered saline (PBS), trypsin from porcine, ethylene-diamine-tetra-acetic acid (EDTA), heat-inactivated fetal bovine serum (FBS), L-glutamine, 2,2-diphenyl-1-picrylhydrazyl (DPPH), absolute ethanol, and (±)-6-Hydroxy-2,5,7,8-tetramethylchromane-2-carboxylic acid (Trolox) were from Sigma-Aldrich (Budapest, Hungary). TNF-α and human IL-1β ELISA Assay Kits were also purchased from Sigma-Aldrich (Budapest, Hungary), while the HaCaT cell line was obtained from Cell Lines Service (CLS, Heidelberg, Germany).

### 2.2. Formulation and Analysis of Self-Emulsifying Drug Delivery Systems

To enhance the absorption of the active compounds in the leaves of the sage, novel drug delivery systems were developed. Self-emulsifying systems were developed by using isopropyl myristate (IPM) as oily phase, nonionic surfactants (Labrafil M 1944 CS and Labrasol) and a co-surfactant (Transcutol HP) in different ratios. A mixture of Labrasol or Labrafil, Transcutol HP, and IPM in the appropriate proportions was prepared at 37 °C using a magnetic stirrer (400 rpm). *S. officinalis* extract at the specified concentration (0.15 *w*/*w*% *S. officinalis* with a carnosol content of 55 µg/mg) was dissolved in the mixture at 24.5 °C under continuous stirring (350 rpm). The final compositions of the self-emulsifying systems are detailed in Table 1. A total of eight formulations, with and without active ingredients, were prepared.

The droplet size of the dispersed phase was determined using the Zetasizer Nano S device (Malvern, UK) for which half a gram of the self-emulsifying system was dissolved in 100 mL of distilled water. The particle size distribution was characterized using the polydispersity index (PDI).

The zeta potential, representing the electrostatic potential of the double layer surrounding the droplets, was also measured using the Zetasizer Nano S device (Malvern Instruments Ltd., Malvern, UK). This analysis provided insights into the stability of self-emulsifying systems. Samples were freshly diluted with 100 mL of distilled water and analyzed in triplicate for each measurement.

To assess the entrapment of sage extract in the formulated SNEDDS/SMEDDS, 100 mg of each formulation was diluted with 100 mL of absolute ethanol. Drug extraction from the SNEDDS/SMEDDS was performed by centrifugation at 10,000 rpm for 20 min. The resulting supernatant was further diluted with absolute ethanol (three times), and the carnosol content was quantified spectroscopically at 280 nm. During UV analysis, the carnosol concentration, which is present in *S. officinalis* extract at 55 µg/mg, was determined. The encapsulation efficiency (EE%) was calculated using the following formula (Equation (1)):

(1) $$Encapsulation\;efficiency\;(\%)\;=\frac{Amount\;of\;carnosol\;measured\;in\;100\;mg\;SNEDDS/SMEDDS}{Amount\;of\;carnosol\;added}$$

The encapsulated drug content (carnosol) was also determined by UV/VIS spectrophotometer at 280 nm.

To investigate the stability of the formulated systems, droplet size, PDI, zeta potential, EE%, and carnosol content were measured 30 and 90 days after formulation, with the samples stored at room temperature (24 °C).

The formulations were tested for thermodynamic stability through heating–cooling cycles, centrifugation, and freeze–thaw cycles. During the heating–cooling cycles, the samples were exposed to six cycles between 4 and 40 °C, with 48 h storage at each temperature. Potential instability manifestations, such as phase separation or precipitation, were visually examined. The formulations were then centrifuged at 2000 rpm for 30 min and examined for phase separation, creaming, or cracking. Finally, the samples underwent three freeze–thaw cycles, alternating between −21 and 40 °C, with 72 h storage at each temperature. The physical appearance of each formulation was visually assessed at the end of each stage [33,34]. After the thermodynamic stability test, the droplet size and polydispersity index were measured to determine the stability of the systems.

The selection of the optimal formulation for incorporation into the cream and gel was based on the evaluation of various physicochemical parameters, including droplet size, PDI, zeta potential, EE%, carnosol content, and stability. The formulation with the most favorable characteristics, demonstrating efficient stability, was chosen for further development into cream and gel formulations. Key parameters considered in the selection process included smaller droplet sizes, which improve bioavailability, as smaller particles are more easily able to penetrate the skin and be absorbed. A low PDI value (e.g., <0.3) was also important, as it indicates a narrower particle size distribution, resulting in a more stable emulsion and better overall formulation quality. Additionally, a high absolute value of the zeta potential (typically > ±30 mV) helped prevent particle aggregation, ensuring long-term formulation stability. The physical and chemical stability of the formulation was crucial, especially in response to temperature variations and mechanical stresses.

### 2.3. Formulation of Topical Dosage Forms

The compositions of the *S. officinalis*-containing creams are presented in Table 2. The oily phase consisting of cetyl stearyl alcohol, stearic acid, glycerin was heated in a water bath at 60 °C. Concurrently, the aqueous phase containing propylene glycol, Cremophor RH 40, and purified water was heated separately. The aqueous phase was then gradually added to the oily phase with gentle stirring to form the cream base. For the Salvia cream, the *S. officinalis* leaf extract was dissolved in IPM, whereas for the Cream + SNEDDS composition, the extract was incorporated in the form of SNEDDS.

The compositions of the formulated gels are presented in Table 3. A predetermined quantity of Carbopol 974P polymer was dispersed in a specified volume of water, followed by neutralization with triethanolamine to initiate gelation. *S. officinalis* extract was either suspended within the prepared gels or incorporated into the formulation as an SNEDDS.

### 2.4. Determination of pH

The pH of the cream/gel was measured by a portable digital pH meter (Mettler Toledo, Zurich, Switzerland). A five-gram sample was dispersed in 20 mL of distilled water heated to 37 ± 2 °C. After 1 min of vigorous stirring and subsequent cooling, the dispersion was filtered, and the pH of the filtrate was determined. This measurement was repeated three times for each sample.

### 2.5. Texture Analysis of Creams and Gels

The textural properties of the formulations were evaluated using a CT3 Texture Analyzer (Brookfield, Middleboro, MA, USA). This instrument is capable of quantifying physical parameters that have been correlated with human sensory perception of topical preparations. Compression tests were conducted. A TA5 Cylinder type probe (35 mm length and 12.7 mm diameter) was employed, with a trigger load of 4.0 g, a target point of 10.0 mm, and a speed of 0.50 mm/s.

Texture Pro CT Software version 1.3 (Brookfield Engineering Laboratories, Middleboro, MA, USA) was used to measure the force required to penetrate and withdraw the probe from the creams and gels. Prior to compression testing, the probe was gently lowered onto the sample surface at a speed of 1 mm/s. Once in contact, the probe penetrated the sample to a depth of 10.0 mm at a speed of 0.50 mm/s, and the applied force was recorded in Newtons (N). Both compression and tension studies were performed in triplicate at a temperature of 24.5 ± 0.5 °C. The test yielded values indicative of the force necessary to penetrate the measuring probe into the cream/gel, providing insights into the textural characteristics of the formulation.

### 2.6. In Vitro Permeation Study

In vitro permeation studies were conducted using Franz diffusion cells (Hanson Microette™ Topical and Transdermal Diffusion Cell System, Chatsworth, CA, USA). For the experiment, a lipophilic surface resembling the skin was created. Cellulose acetate synthetic membranes were pretreated by soaking them in IPM for 10 min. This synthetic membrane allows for a skin-mimicking environment to assess the permeation of the active compound. The diffusion area of the membranes was 1.767 cm^2^. To ensure sink conditions, the receptor medium (1 mL) consisted of a 1:1 mixture of ethanol (70% *v*/*v*) and pH 7.4 phosphate-buffered saline (PBS). This solvent system was chosen based on its ability to provide sufficient solubility for the active compound. This approach guarantees that the drug remains in a state of constant diffusion without reaching saturation, thereby allowing accurate measurement of its permeation during the experiment. The receptor phase was kept at 32.5 ± 0.5 °C with a magnetic stirrer set to 350 rpm.

In the permeation experiment, for each formulation, 1.0 g containing 15 mg of *S. officinalis* extract (equivalent to 825 µg of carnosol) was applied to the membrane. At predetermined intervals (15, 30, 60, 90, and 120 min), 1 mL aliquots were withdrawn from the receptor phase. To maintain constant volume in the receptor phase, fresh receptor solution (1 mL) was added after each sample withdrawal.

Sample absorbance was measured using a UV spectrophotometer (Shimadzu, Tokyo, Japan) at 280 nm to determine carnosol concentration [35]. A blank sample of the ethanol (70% *v*/*v*) and PBS mixture (1:1) was used as a reference, and a calibration curve for carnosol was created prior to measurement. For the calibration curve, a series of standard solutions with carnosol concentrations of 1, 5, 10, 20, 50, 100, 150, and 200 µg/mL were prepared. These concentrations were chosen to cover a wide range of expected values in the receptor solution, ensuring accurate measurement of carnosol levels. The calibration curve was constructed by plotting the absorbance values of these standards, with a determination coefficient (R^2^) of 0.9994, indicating excellent linearity and reliability of the calibration process.

To analyze and compare the permeation profiles of cream or gel formulations, both with and without self-emulsifying systems, difference factor (*f*1) and similarity factor (*f*2) were calculated. The difference factor quantifies the percent variation between two profiles at each data point, whereas the similarity factor assesses the closeness between the two profiles. Equations (2) and (3) were applied to compute the values of *f*1 and *f*2 [36,37].

(2) $$f_{1}=\left[\frac{\Sigma (R_{t}-T_{t})}{\Sigma R_{t}}\right]\times 100$$

(3) $$f_{2}=50\times log\left[\left\{\frac{1+\left(R_{t}-T_{t})\times 1\right.}{n}\right\}-0.5\right]$$

where *n* represents the sampling number, while *R_t_* and *T_t_* denote the percentages of dissolved reference and test formulations at each time point *t*. Two dissolution profiles are considered to be similar and bioequivalent when the difference factor (*f*1) falls between 0 and 15, and the similarity factor (*f*2) is between 50 and 100.

The release rate (k) of carnosol was ascertained from the slope of the amount of drug released per unit area (µg/cm^2^) versus the square root of time (min½). The diffusion coefficient (*D*, cm^2^/h) of carnosol was determined using the cumulative amount of carnosol that diffused per unit area at a given time (*Q*, μg/cm^2^), the initial concentration of carnosol in the formulation (*C*_0_′, μg/cm^3^) and the diffusion time (*t*, h). This calculation is derived from Fick’s second law of diffusion, which describes the relationship between the diffusion coefficient, the cumulative amount of drug diffused, and time. Specifically, the equation used (Equation (4)) relates the diffusion coefficient to the cumulative drug concentration at time *t*, the initial drug concentration in the donor phase (*C*_0_′), and the elapsed time, thereby quantifying the rate of diffusion across the membrane.

(4) $$D=\frac{Q^{2}\times \pi }{(2[C_{0}^{\prime }{])}^{2}\times t}$$

### 2.7. Cell Culturing

The immortalized human keratinocyte line HaCaT, which exhibits functional characteristics comparable to normal keratinocytes, was utilized in the following in vitro examinations. HaCaT cells were cultured in DMEM supplemented with 3.7 g/L NaHCO_3_, 10 *v*/*v*% FBS, 1 *v*/*v*% nonessential amino acids solution, 1 *v*/*v*% L-glutamine, 100 IU/mL penicillin, and 1001 IU/mL streptomycin. The culture was maintained at 37 °C in a humidified atmosphere containing 5% CO_2_. The culture medium was refreshed twice weekly, and cells were propagated through regular passaging. For the in vitro cytotoxicity and anti-inflammatory assays, HaCaT cells within the 20 to 30 passages were employed.

### 2.8. In Vitro Citotoxicity Assay

The cytotoxicity of the preparations was evaluated using the MTT assay, a colorimetric method that measures mitochondrial dehydrogenase activity [38]. This enzyme converts the yellow MTT dye to a dark blue formazan crystal [(E,Z)-5-(4,5-dimethylthiazol-2-yl)-1,3-diphenylformazan] upon reduction [39].

HaCaT cells were seeded onto 96-well plates at a density of 10^4^ cells/well and incubated at 37 °C for 4 days. After removing the culture medium, test solutions (prepared by mixing the formulated creams and gels with PBS at a concentration of 0.1, 0.5 and 1 *w*/*v*%) were added and incubated for 2 h. Following a PBS wash, 0.5 mg/mL MTT solution was added, and the cells were incubated for an additional 3 h. Finally, 0.1 mL acidic isopropanol solution (isopropanol: 1.0 M hydrochloric acid = 25:1) was added to dissolve the formed formazan crystals. Absorbance was measured at 570 nm with a reference wavelength of 690 nm reference using a Multiskan GO Microplate Spectrophotometer (Thermo Fisher Scientific Oy, Ratastie, Finland). Cell viability was calculated as a percentage compared to control cells treated with PBS. The absorbance values directly correlated with the number of viable cells. The test was performed with six wells per sample.

### 2.9. DPPH Radical Scavenging Activity

The antioxidant capacity of the diluted samples (5% *w/v* absolute ethanol) was evaluated using the DPPH (C_18_H_12_N_5_O_6_, M = 394.33 g/mol) free radical method [40]. A solution of 0.06 mM 2 mL DPPH in absolute ethanol was combined with 900 µL absolute ethanol, followed by the addition of 100 µL of the sample. The mixture was incubated at room temperature for 30 min, during which time antioxidant compounds in the sample reacted with DPPH, resulting in a color change from deep purple to light yellow [41].

The remaining DPPH concentration was measured UV-spectrophotometer at 517 nm [42]. Absolute ethanol served as the blank control, while Trolox (0.5 *w*/*v*%) and a 2 mL DPPH solution diluted with absolute ethanol were used as positive and negative controls, respectively. The antioxidant activity percentage (AA% = antioxidant activity) was calculated according to the method described by Mensor et al. [43]:

(5) $$AA\%=100-\left.\left[\frac{({Abs}_{sample}-{Abs}_{blank})\times 100}{{Abs}_{control}}\right]\right.$$

### 2.10. In Vitro Anti-Inflammatory Effect

To evaluate the in vitro anti-inflammatory effects of the topical formulations, human TNF-α (Sigma—RAB0476) and IL-1β (Sigma—RAB0273) ELISA tests were conducted on the HaCaT cell line in 96-well plates (10^4^ cells/well). The test solutions were prepared by mixing the formulated creams and gels with PBS at a concentration of 1 *w*/*v*%. From each sample, 200 μL was added to each well and incubated for 1 h. IL-6 proinflammatory cytokine was used to induce inflammation in keratinocyte cells. At the end of the incubation period, the test solutions were removed, and 50 μL of IL-6 (20 ng/mL) was added to each well, followed by incubation for 24 h at 37 °C in a 5% CO_2_ atmosphere. Subsequently, the culture supernatants were collected for TNF-α and IL-1β quantification using a commercially available ELISA kit according to the manufacturer’s instructions.

### 2.11. Experimental Animals

Female *Rattus norvegicus* (SPRD) rats (Charles River Laboratories International, Inc., Sulzfeld, Germany), with an average weight of 546 ± 34 g, were used in the in vivo experiments. The inclusion criteria required healthy animals with no visible signs of illness or injury, and they were of similar age and weight to ensure consistency. Exclusion criteria included animals exhibiting any signs of illness, injury, or abnormalities that could affect the study outcomes. The rats were kept and treated in accordance with the “Principles of Laboratory Animal Care” established by the National Society for Medical Research, and the “Guide for the Care and Use of Laboratory Animals” by the National Academy of Sciences, published by the National Institutes of Health. The animals were kept in wire-bottomed cages, maintained on a 12:12 h light–dark cycle and provided with standard rodent chew pellets and water ad libitum throughout the study. Each formulation intended for testing (positive, negative controls, and Cream + SNEDDS) was evaluated on six animals. The sample size was determined based on the availability of resources and ethical considerations. Animals were randomly assigned to control and treatment groups to minimize bias and ensure comparable baseline characteristics across all groups. Randomization was achieved using a simple random number generator. Approval number: 10/2022/DEMÁB.

### 2.12. Carrageenan-Induced Acute Inflammatory Model

The anti-inflammatory activity was assessed through the carrageenan-induced paw edema test in rats. Each composition was tested on eight animals. In the in vivo experiments, the rats were anesthetized using a combination of ketamine and xylazine at doses of 50 and 10 mg/kg body weight, respectively [44]. The thickness of the paws was measured using an engineering precision caliper, and both the outer and inner surfaces of their left front paw were pretreated with the selected creams. In this study, methylprednisolone aceponate cream (1 *w*/*w*%) was used as the positive control, while the formulated cream without *S. officinalis* (base cream) served as the negative control.

Edema was triggered by administering a 100 μL subplantar injection of a 1% carrageenan solution into the left forepaws. Paw thickness was recorded immediately before the injection (“0 h”), 3 h and 24 h afterward. The increase in paw thickness was determined by calculating the difference between the measurements at “0 h” and 3 h and 24 h post-injection.

After measuring paw thickness, the animals were euthanized with pentobarbital injection (100 mg/kg body weight). The treated skin surface was isolated, and the tissue lysates were homogenized with 500 μL of sterile saline, followed by centrifugation (1500× *g*, 15 min, 4 °C). The supernatants were then used to determine the levels of proinflammatory cytokines. For the determination of TNF-α and IL-1β, ELISA kits (Sigma Aldrich Kft.) were used according to the manufacturer’s instructions.

### 2.13. Statistical Analysis

All data were analyzed using GraphPad Prism (version 10.4; GraphPad Software, San Diego, CA, USA) and are herein presented as means ± SD. To compare the outcomes of the texture analysis, in vitro cell viability, and antioxidant and anti-inflammatory assays, as well as the in vivo experimental results, one-way ANOVA was applied, followed by Dunnett’s or Tukey’s post hoc multiple comparison tests. The difference in means was considered significant in the case of *p* < 0.05.

## 3. Results

### 3.1. Evaluation of the Formulated Self-Emulsifying Drug Delivery Systems

The diameter of the dispersed phase was analyzed using the Zetasizer NanoS device, which also provided measurements of droplet size, zeta potential, and polydispersity index (PDI) (Table 4). It was found that formulations with higher surfactant and co-surfactant content (I_0_, I, III_0_, III) exhibited droplet sizes below 100 nm, classifying them as self-nanoemulsifying drug delivery systems (SNEDDSs). In all cases, formulations containing the active ingredient displayed larger droplet sizes; however, the size-increasements were not significant. Results also indicated that formulations with higher amounts of the oily phase had significantly larger droplet sizes compared to those with lower IPM content. Specifically, formulations II, II_0_, IV, and IV_0_ exhibited droplet sizes in the range of 0.2–0.4 μm, which aligns with the literature definition of self-microemulsifying drug delivery systems (SMEDDSs) [24,27]. These findings suggest that the droplet size of the dispersed phase is strongly influenced by the composition of the formulation, especially by the amount of surfactants.

It was observed that all formulations were monodisperse systems, with zeta potential values ranging from −15.07 ± 0.29 mV to −29.54 ± 0.48 mV. All compositions exhibited a negative electrical charge, which may be due to the electrical charge of the emulsifier molecules. The stability of the systems improved with higher absolute zeta potential values, as the increased surface charge reduced particle aggregation. Based on the zeta potential measurements, SNEDDSs exhibited significantly greater stability compared to SMEDDSs. Furthermore, the addition of the active ingredient did not significantly influence the zeta potential.

The EE% and the results of the measurement of the carnosol content presented in Table 4 offer valuable insights into the performance of the different self-emulsifying drug delivery system formulations regarding active ingredient entrapment. The EE% indicates the proportion of the active compound successfully incorporated into the carrier system relative to the total amount used in the formulation. The encapsulation efficiency of SNEDDS formulations (93.12 ± 1.27% for I and 95.73 ± 1.51% for III) was significantly higher than that of SMEDDS formulations (82.66 ± 1.86% for II and 85.03 ± 1.33% for IV). In line with this, a carnosol concentration exceeding 50 µg/mg was measurable in the SNEDDS formulations, further demonstrating their superior ability to incorporate the active ingredient effectively. This suggests that SNEDDSs were more effective in encapsulating the active compound, likely due to their smaller droplet size and higher surfactant concentration, which improves solubilization and stability. SMEDDSs with larger droplets and lower surfactant content showed lower encapsulation efficiency. However, both formulation types displayed consistent drug loading across samples, indicating uniform distribution of the active compound.

To investigate the stability of the formulated systems, these parameters (droplet size, PDI, zeta potential, EE%, and carnosol content) were also measured after 30 and 90 days. The results of these stability measurements were presented in Table 5 and Table 6.

Based on the obtained results, it can be concluded that our self-emulsifying systems remained stable even after 90 days of storage at room temperature. No significant differences were detected in droplet size, zeta potential, PDI, encapsulation efficiency, or active ingredient concentration during this period.

The results of the thermodynamic stability tests demonstrated that the formulations with a larger droplet size (SMEDDS) exhibited phase separation following heating–cooling cycles, centrifugation, and freeze–thaw cycles, as summarized in Table 7. Visual inspection confirmed that the physical appearance of self-emulsifying systems with lower particle size remained unchanged after the tests. These findings indicate that the thermodynamic stability of composition I_0_, I, III_0_, and III were satisfactory.

After the thermodynamic stability test, the droplet size and polydispersity index were measured to confirm the stability of the formulated systems. The results presented in Table 8 correlated with the visual inspection; there were significant changes in droplet size and PDI values in the case of formulations II_0_, II, IV_0_, and IV. The high PDI for formulations II_0_, II, IV_0_, and IV suggests a broad particle size distribution, indicating significant variation in particle sizes within the systems.

Following the evaluation and comparative analysis of the results obtained from the investigation of self-emulsifying systems, composition III was identified as the optimal candidate for the formulation of topical drug delivery systems as the zeta potential of this composition approaches −30 mV. This formulation incorporated Labrafil M as a surfactant, and its droplet size, upon dispersion in an aqueous medium, was determined to fall within the nanometer range, thus qualifying it as an SNEDDS.

These results suggest that SNEDDSs may be more suitable for lipophilic drug delivery.

### 3.2. pH Measurement of Topical Formulations

The aim during the preparation of both creams and gels was to achieve a pH close to that of the skin’s natural surface, approximately 4.7; however, topical formulations that have the pH range of 4–7 are accepted [45]. Table 9 presents the pH values of different topical formulations at three time points. The pH value of the compositions was between 5.75 and 6.58 immediately after formulation.

In the case of gels, achieving the ideal pH is particularly challenging due to the necessity of maintaining a pH close to neutral to initiate the gelling process. To ensure this, triethanolamine (TEA) was used as a pH adjuster during the formulation process. The addition of TEA was crucial for activating the gelling agent (Carbopol 974P) and ensuring the proper consistency of the gel. This explains the higher pH values observed for the gel formulations, as the pH was adjusted to an optimal level for the gelation process while remaining within an acceptable range for topical application.

### 3.3. Texture Analysis

**Figure 1:**
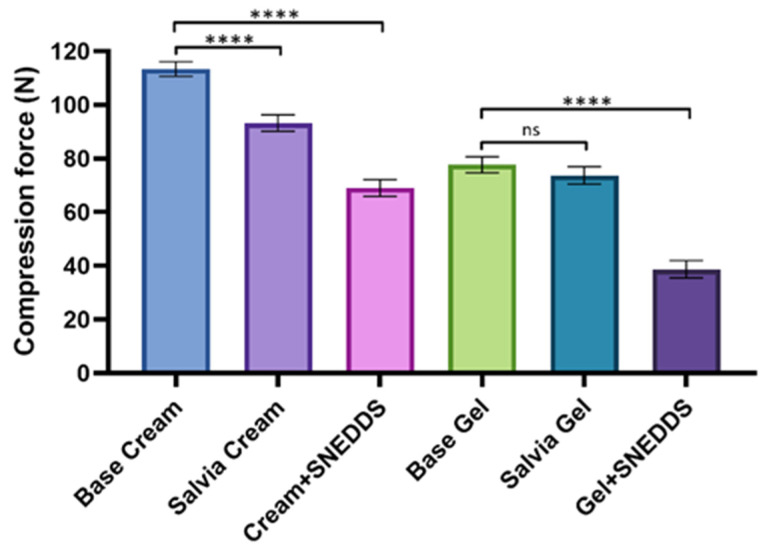
Texture analysis of creams and gels at 25 °C, measured as compression force. Data are presented as mean ± SD (*n* = 6). Statistical comparisons between different formulations and the base cream/gel were performed using one-way ANOVA with Dunnett’s multiple comparison test. **** indicate statistically significant, *p* < 0.0001 and “ns” means not significant.

Figure 1 illustrates the compression force values of various formulations, which are indicative of the firmness or hardness of the product texture. The formulations containing *S. officinalis* extract, both with and without an SNEDDS, were compared to the base formulations (cream/gel without active ingredient). The base cream exhibited the highest compression force, suggesting that it is the firmest or hardest formulation. Both the Salvia cream and the Cream + SNEDDS formulations displayed significantly lower compression forces, which likely reflect a softer texture or reduced resistance. The base gel and Salvia gel demonstrated similar compression forces, both significantly lower than the base cream. The Gel + SNEDDS formulation exhibited the lowest compression force, indicating the softest texture.

### 3.4. In Vitro Permeation Study

**Figure 2:**
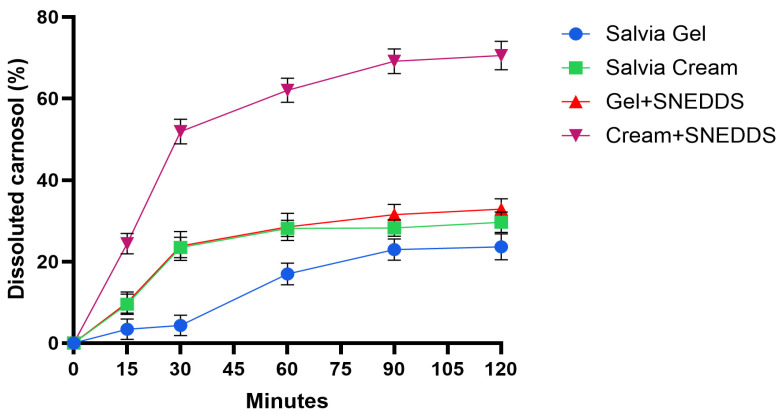
The in vitro release profiles of carnosol from the gels and creams. Bars represent the mean ± SD, *n* = 6.

In vitro permeation profiles of the creams and gels formulations were investigated using the Franz diffusion method. Figure 2 illustrates the percentage of permeated carnosol over time for different formulations across IPM-impregnated cellulose acetate membrane. The highest rate and overall percentage of carnosol dissolution has been observed in the Cream + SNEDDS formulation, reaching nearly 80% by the end of the test period. This increased dissolution has likely been facilitated by the presence of an SNEDDS, which enhances the solubility and bioavailability of active compounds by creating a fine emulsion. Although the Gel + SNEDDS formulation also contains the SNEDDS, its rate and total percentage of dissolved carnosol have been lower than the Cream + SNEDDS. This difference may have been caused by the gel base, which could have restricted the carnosol diffusion compared to the cream. Creams generally contain more oils and emollients, which may have better facilitated the release of active ingredients when combined with SNEDDS. A moderate carnosol dissolution rate has been observed in the Salvia cream without SNEDDS, leveling off much earlier than the Cream + SNEDDS. However, some level of carnosol dissolution has still been supported by the cream base due to its formulation properties.

The lowest carnosol dissolution has been seen in the sage gel, with only a minimal dissolution by the end of the test period. The gel formulation may have limited carnosol release, as gels are typically more water-based and lack the lipid-rich environment of creams, which can help to solubilize certain compounds.

Upon calculating the diffusion coefficients, it was observed that the Cream + SNEDDS exhibited the fastest carnosol diffusion, with a value of 0.19448 ± 0.01834 cm^2^/h (Table 10). In contrast, the same formulation without an SNEDDS had a diffusion coefficient of only 0.04056 ± 0.00266 cm^2^/h. Additionally, it was noted that the release rate and diffusion coefficient of the formulations without an SNEDDS were very similar. Notably, no significant differences were detected between the gel formulations.

The diffusion profiles of compositions with and without an SNEDDS were compared. The calculated difference factors (*f*_1_) and similarity factors (*f*_2_) are presented in Table 11. The results revealed significant differences in the diffusion profiles of carnosol between compositions with and without SNEDDS, as all *f*1 values exceeded 15 and all *f*2 values were below 50. However, for Sage gel versus Gel + SNEDDS, the *f*2 value closely approached 50, suggesting a smaller difference between these two formulations compared to the creams.

### 3.5. In Vitro Citotoxicity Assay

**Figure 3:**
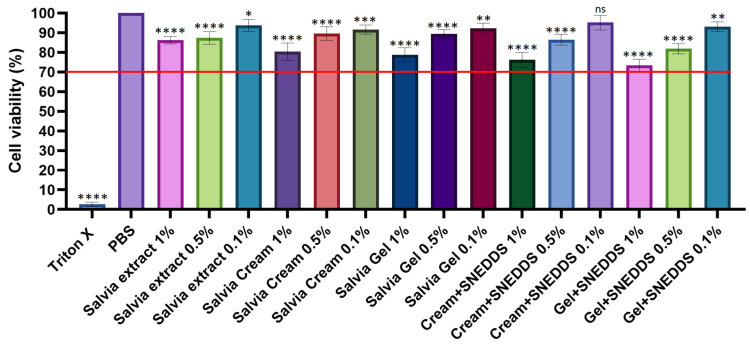
Cell viability of HaCat cells incubated with the formulations for 2 h. Viability is expressed as a percentage of the negative control (PBS). Data are presented as mean ± SD (*n* = 6). Ordinary one-way ANOVA with Dunnett’s multiple comparison test was performed to compare the different formulations with PBS. Statistically significant differences are indicated by *, **, ***, and **** for *p* < 0.05, *p* < 0.01, *p* < 0.001, and *p* < 0.0001, respectively, while “ns” indicates no significant difference. The red line represents the threshold for cytotoxicity level of 70% of cells viability compared to the negative control.

The results of the MTT test are presented in Figure 3. In the experiment, PBS was used as a negative control and Triton X-100 was used as a positive control. Cell viability values were compared to those for PBS and expressed as a percentage of the negative control. The Salvia extract at 1%, 0.5%, and 0.1% concentrations showed cell viability between 80% and 95%, indicating relatively low cytotoxicity at these concentrations. The Salvia cream maintained consistently high cell viability, with slight but significant differences between concentrations. Similarly, the Salvia gel demonstrated comparable results to the cream, maintaining cell viability above 70% with minor variations across concentrations.

Formulations with SNEDDSs at 1% concentration generally showed lower cell viability compared to the 0.5% and 0.1% concentrations, but it remained above the threshold. Among all formulations, the 0.1% Cream + SNEDDS exhibited the highest cell viability, approximately 90%, which was also the highest value among the SNEDDS formulations. These results suggest that Salvia-based preparations, especially in combination with SNEDDSs, are safe and biocompatible. Differences between the various formulations and concentrations are statistically significant, but cell viability generally remains above 80%.

### 3.6. DPPH Radical Scavenging Activity

**Figure 4:**
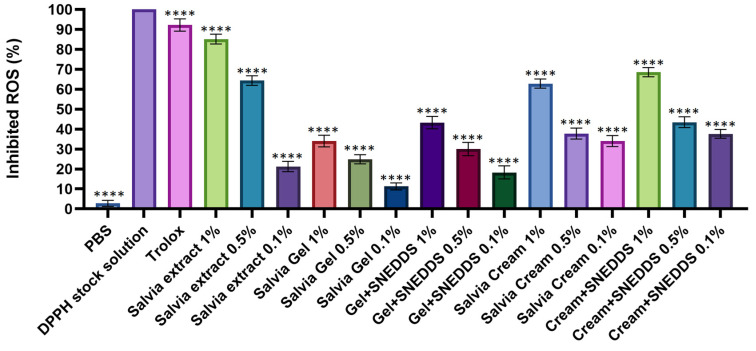
DPPH-scavenging activity of the *S. officinalis* extract and the formulations. Data are presented as mean ± SD (*n* = 6). An ordinary one-way ANOVA with Dunett’s multiple comparison test was performed to compare the extracts and the different formulations with PBS. **** indicate statistically significant differences at, *p* < 0.0001.

The percentage of antioxidant activity (AA%) of the four formulations and their dilution series was determined using a DPPH test solution. PBS was employed as the negative control, while Trolox (0.5 *w*/*v*%) was used as the positive control, showing the highest inhibition of reactive oxygen species (ROS). The results of the experiment are presented in Figure 4. The 1% and 0.5% concentrations of sage extract exhibited remarkably high ROS inhibition. In particular, the 1% concentration achieved an 85% ROS inhibition, representing the highest observed value. The Salvia gels showed moderate antioxidant activity, with the 1% gel exhibited the highest efficacy (~30%), while the 0.5% and 0.1% gels exhibited lower activity. The Gel + SNEDDS formulations demonstrated improved antioxidant activity compared to the Salvia gel alone, with the 1% Gel + SNEDDS formulation showing ~45% ROS inhibition, higher than the other concentrations.

Salvia creams displayed antioxidant activity similar to the gels, with the 1% concentration exhibiting the greatest efficacy (~60%). The Cream + SNEDDS formulations exhibited the highest ROS inhibition among the creams, with the 1% Cream + SNEDDS formulation exceeding 70%, which is comparable to the antioxidant effect of the Salvia extract.

Formulations containing an SNEDDS enhanced the antioxidant effect compared to the corresponding gel or cream without an SNEDDS. Across all types of formulations, higher concentrations (1%) generally provided stronger antioxidant effects. The 1% Cream + SNEDDS formulation showed the most potent antioxidant activity, highlighting the synergistic effect of Salvia and an SNEDDS in combating ROS.

### 3.7. Examination of In Vitro Anti-Inflammatory Effect

**Figure 5:**
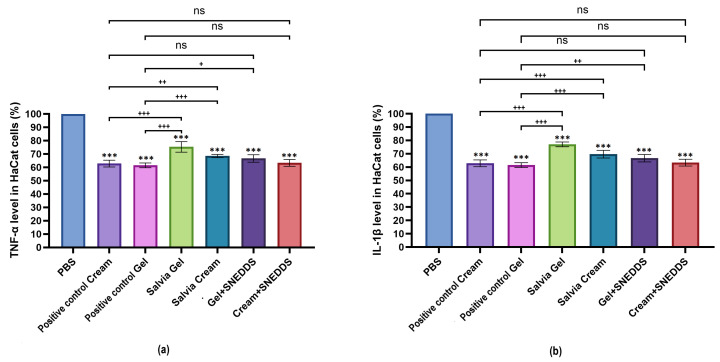
Results of human TNF-α (**a**) and IL-1β (**b**) ELISA tests on HaCaT cells. Ordinary one-way ANOVA with Dunnett’s multiple comparison test was performed to compare the different formulations with PBS (signed with *), and with the positive controls (signed with +). The *** indicates statistically significant differences at *p* < 0.05, while +, ++, and +++ indicate statistically significant differences at *p* < 0.05, *p* < 0.01, *p* < 0.001, and *p* < 0.0001 and “ns” means not significant.

The in vitro anti-inflammatory effect of the preparations was investigated using ELISA tests on HaCaT cell lines. PBS was chosen as a negative control, showing 100% TNF-α levels, which served as a baseline for comparison with other treatments. As shown in Figure 5a,b samples containing *Salvia officinalis* reduced both proinflammatory cytokines to a similar extent. There was no significant difference between the methylprednisolone aceponate preparations (used as a positive control) and the sage preparations containing SNEDDSs. Among the formulations developed by us, the Cream + SNEDDS preparation exhibited the strongest anti-inflammatory effect on HaCaT cells. Furthermore, it was evident that the inclusion of *S. officinalis* enhanced the anti-inflammatory effect in all treatments.

### 3.8. Rat Paw Edema Test

**Figure 6:**
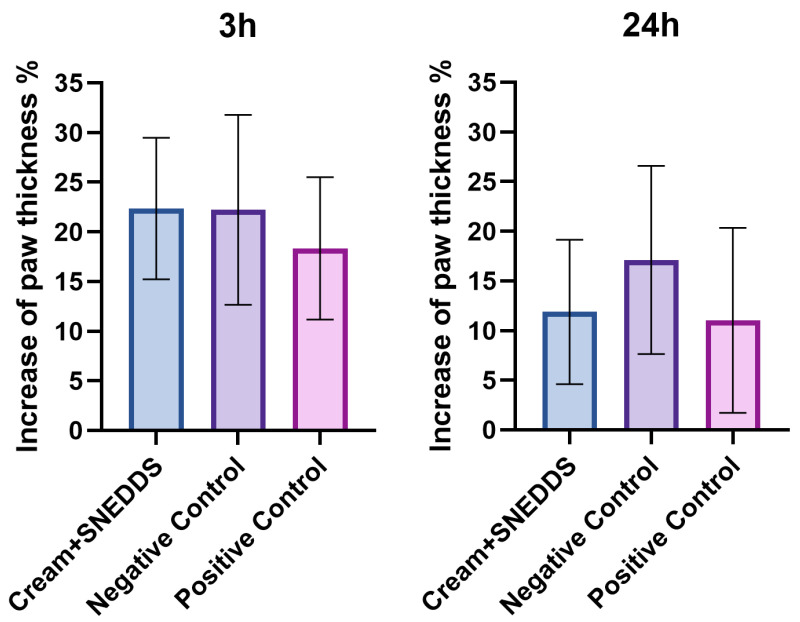
Evaluation of Cream + SNEDDS on carrageenan-induced rat paw edema at 3 h and 24 h. Data are presented as mean ± SD, *n* = 6. Positive control: methylprednisolone aceponate cream (1 *w*/*w*%); Negative control: cream without *S. officinalis* extract (base cream).

Figure 6 illustrates the increase in paw thickness (%) at 3 h (3 h) and 24 h (24 h) after inducing edema in rat paws using carrageenan injection. The paw thickness was measured to evaluate the anti-inflammatory effects of the tested Cream + SNEDDS formulation in comparison to a negative control (untreated group) and a positive control (treated with a known anti-inflammatory agent, methylprednisolone aceponate).

At the 3 h mark, all groups, including the Cream + SNEDDS, negative control, and positive control, show similar levels of paw swelling, suggesting the early stage of inflammation was not significantly mitigated.

By 24 h, the paw swelling is reduced in all groups. Notably, the Cream + SNEDDS group exhibits a decrease in swelling comparable to the positive control, indicating potential anti-inflammatory activity. The negative control group maintains a higher paw thickness compared to the treated groups, highlighting the natural progression of inflammation without treatment.

According to the results of the measurement of TNF-α and IL-1β levels, the cream containing sage extract incorporated to an SNEDDS (Cream + SNEDDS) showed significantly lower cytokine levels in both cases compared to the negative control (PBS) (Table 12). Formulations without the extract did not influence the production neither TNF-α, nor IL-1β. Comparing the levels of TNF-α and IL-1β, it was found that the amount of TNF-α decreased more significantly as the result of the treatment with the Cream + SNEDDS. Furthermore, our formulations were able to exceed the anti-inflammatory effect of the steroid-containing cream in the case of carrageenan-induced inflammation.

## 4. Discussion

The present study describes the development of novel *S. officinalis*–containing formulations and the investigation of their antioxidant and anti-inflammatory potential. As previously noted, topically applied products containing natural active compounds are playing an increasingly important role in treating various inflammatory skin conditions. While the external use of *S. officinalis* is not yet widespread, it holds valuable ingredients known for their antioxidant and anti-inflammatory properties [7,8,11]. The bioactive compounds in sage, especially carnosol, are increasingly being incorporated into topical formulations due to their ability to enhance skin health. Carnosol, through its antioxidant and anti-inflammatory activities, helps to protect the skin from oxidative damage, reduce inflammation, and support skin regeneration [20,21]. These properties make sage-based formulations, such as creams, gels, and oils, effective in treating conditions like acne, eczema, and other inflammatory skin diseases. However, the water solubility of carnosol is quite limited, which significantly restricts its bioavailability and absorption when used in topical formulations. This low solubility in aqueous environments poses a challenge for its effective incorporation into formulations that require efficient delivery of the active compound to the skin. As a result, the therapeutic potential of carnosol may be reduced unless suitable formulation strategies, such as the use of carriers like self-nanoemulsifying drug delivery systems (SNEDDSs), are employed to enhance its solubility and bioavailability [30,31,32].

The evaluation of self-emulsifying drug delivery systems (SEDDSs) and their application in topical formulations has garnered significant interest in pharmaceutical research. The findings of this study align with and expand upon existing literature, offering insights into the interplay between formulation composition, stability, and functional properties.

In accordance with earlier studies, formulations with higher surfactant and co-surfactant content (e.g., compositions I_0_, I, III_0_, and III) exhibited droplet sizes below 100 nm, classifying them as self-nanoemulsifying drug delivery systems (SNEDDSs). The smaller droplet sizes observed in certain formulations, particularly those with higher surfactant and co-surfactant content, are likely a result of the increased emulsification efficiency facilitated by the surfactants. Surfactants reduce the interfacial tension between the oil and aqueous phases, promoting the formation of smaller droplets. The choice of surfactant and co-surfactant, as well as their concentration, plays a significant role in controlling the droplet size, as higher surfactant content typically leads to finer emulsions [46]. In the present study, it was found that the combination of Labrasol/Labrafil M, Transcutol HP, and IPM in a 6:6:5 ratio resulted in smaller, more uniform droplet sizes, which are highly desirable for drug delivery applications. Smaller droplet sizes, as observed, enhance the dissolution and bioavailability of lipophilic drugs like carnosol by increasing the surface area available for absorption. This agrees with studies by Pouton, which highlighted the critical role of droplet size in drug solubilization and absorption enhancement [47]. The uniform droplet size distribution is also crucial for formulation stability, with PDI values ranging from 0.050 to 0.700 generally being acceptable for most formulations, indicating a balance between homogeneity and acceptable drug release [48]. According to our results the formulated systems were monodispersed with PDI values between 0.169 and 0.217.

The zeta potential values measured (ranging from −15.07 mV to −29.54 mV) indicate that all formulations were sufficiently stable, with SNEDDS formulations demonstrating superior stability. High absolute zeta potential values prevent droplet aggregation through electrostatic repulsion, corroborating the findings of previous studies, which emphasized the importance of zeta potential in maintaining colloidal stability [49,50] The higher absolute zeta potential values, particularly in SNEDDS formulations, can be attributed to the electrostatic repulsion between the charged droplets. This electrostatic charge is primarily influenced by the type and concentration of surfactant used. Surfactants are typically designed to impart a negative or positive charge to the droplets, which prevents aggregation by creating a repulsive force between similarly charged particles. In our case, the higher surfactant concentrations likely led to a higher charge density on the droplets, enhancing their stability by preventing aggregation [49,50,51]. Moreover, the stability of SNEDDS formulations could be further attributed to the robustness of the surfactant system. The high zeta potential values suggest that the surfactants used in these systems were effective at maintaining electrostatic repulsion, preventing particle aggregation and promoting long-term stability. The lack of significant alteration in zeta potential values upon the addition of carnosol further supports the robustness of the surfactant systems, indicating that the active components did not interfere with the overall formulation stability [42,51,52].

The thermodynamic stability of self-emulsifying systems is also a critical parameter for their development and practical application in drug delivery. These tests are pivotal in evaluating the robustness of formulations and their suitability for long-term storage and practical applications [53,54]. The results of the current study revealed that formulations with smaller droplet sizes exhibited superior stability under heating–cooling, centrifugation, and freeze–thaw stress tests. Formulations with larger droplet sizes (SMEDDS) showed phase separation under stress, indicating their limited thermodynamic stability. Previous studies have documented similar findings, attributing instability to the higher oil content and insufficient surfactant-to-oil ratios in SMEDDS, which can lead to inadequate stabilization of the dispersed phase [30]. Our results extend this understanding by demonstrating that such formulations are particularly vulnerable to temperature fluctuations and mechanical stress.

Consistent with findings reported in previous studies, smaller droplet sizes in SNEDDS formulations are associated with enhanced stability due to improved emulsification efficiency and reduced likelihood of coalescence. The literature indicates that formulations with droplet sizes below 100 nm and high absolute zeta potential values (e.g., approaching or exceeding −30 mV) are less susceptible to aggregation, providing greater thermodynamic stability under stress conditions [55,56]. Our results align with these observations, as composition III, with a zeta potential of −29.54 ± 0.48 mV and a droplet size of 51.14 ± 2.74 nm, remained stable. Therefore, this composition was selected for the formulation of cream and gel.

The formulated creams and gels with or without an SNEDDS were analyzed for consistency, dissolution profile, biocompatibility, and for antioxidant and anti-inflammatory activity. Texture analysis revealed that creams had higher firmness. As Bolla et al. described, the choice of vehicle in topical formulations can significantly influence the bioavailability of active ingredients [57]. One key factor is the texture of the formulation, as it can affect the release and diffusion of the drug from the vehicle. Gels, with their softer and more fluid texture, tend to allow for faster drug diffusion, resulting in a quicker release. However, in our study, the cream formulation exhibited better drug release and permeation despite its more solid texture. This could be due to the unique characteristics of the lipid-rich matrix, which may provide a more favorable environment for the sustained release of lipophilic compounds, ensuring prolonged skin contact and better penetration. The observed decrease in texture hardness upon incorporation of an SNEDDS may indicate a softer, more easily spreadable formulation, a feature that is often desired in topical products to improve patient compliance and ease of application. The softer texture observed in formulations containing an SNEDDS also correlated with previous studies, which report that nanoemulsion-based systems tend to reduce formulation viscosity, thereby enhancing spreadability and patient compliance [58,59,60].

The enhanced permeation of carnosol from Cream + SNEDDS formulations, reaching nearly 80%, underscores the potential of SNEDDSs in improving the solubility and bioavailability of lipophilic actives. This finding is consistent with several studies, including those by Bravo-Alfaro et al. and Mohite et al., which demonstrated that SNEDDSs significantly enhance the bioavailability of active compounds by effectively solubilizing poorly water-soluble drugs [33,61]. Similarly, van Staden et al. reported that SNEDDS formulations can improve drug permeation and absorption due to their nanosized droplets and enhanced surface area [49]. The enhanced permeation rates of the cream containing the nano-system can be attributed to the nanosized droplet size and the improved stability provided by the SNEDDS. Smaller droplet sizes significantly increase the surface area for drug absorption, facilitating deeper penetration of the active ingredient through the skin [62]. Additionally, the high zeta potential of the formulation helps prevent aggregation, ensuring that the drug remains in a stable and efficient form for permeation [63]. These properties combined lead to better bioavailability and improved therapeutic effects, as observed in the in vitro and in vivo tests conducted in this study. This suggests that the incorporation of the SNEDDS into the cream formulation enhances both the solubility and the skin penetration of the active compound, offering a promising delivery system for topical drug applications. The lower dissolution rate observed in the Gel + SNEDDS formulation is likely due to the water-based nature of gels, which typically provide a less lipid-rich environment for drug diffusion compared to creams. Previous studies support this observation, indicating that the oil-based matrix of creams promotes superior drug release compared to gel formulations due to better compatibility with lipophilic drugs [64,65,66]. These findings emphasize the critical role of formulation bases in influencing drug permeation.

The high cell viability observed for most formulations, particularly at concentrations of 0.1%, 0.5%, and 1%, indicates that the sage-based formulations, including those incorporating SNEDDSs, are biocompatible and exhibit minimal cytotoxicity. These findings are consistent with earlier research by Abu-Darwish et al., which demonstrated that *S. officinalis* exhibited low cytotoxicity at therapeutic concentrations (0.64 μL/mL). This study also supports the notion that sage-based formulations are safe for topical use and biocompatible, making them suitable for treating inflammatory skin conditions [67].

The findings from the DPPH and ELISA assays suggest that these formulations may provide significant protection against oxidative stress and inflammation, both of which are key contributors to skin aging and other dermatological conditions. The significant antioxidant activity (up to 85% ROS inhibition) and anti-inflammatory effects observed for sage-based formulations, particularly those with SNEDDSs, correlated with the reported pharmacological properties of *S. officinalis* [16,68,69]. The enhanced efficacy of Cream + SNEDDS formulations compared to gels can be attributed to the lipid-rich environment of creams, which facilitates the release of lipophilic compounds like carnosol. The superior anti-inflammatory effects of the Cream + SNEDDS formulation are particularly promising, as this formulation could be a potential treatment for inflammatory skin conditions such as dermatitis and psoriasis.

The carrageenan-induced paw edema model is commonly employed to evaluate the anti-inflammatory activity of various natural and synthetic compounds [70]. Our in vivo anti-inflammatory results further supported the beneficial effects of the SNEDDS-containing cream, which exhibited a significant reduction in paw edema following carrageenan injection. While it did not outperform methylprednisolone aceponate, the lack of a significant difference suggests that this formulation may be a viable alternative for the treatment of acute inflammation. Maione and his research team also reported that carnosol exhibited notable and dose-dependent anti-inflammatory and anti-nociceptive effects in mouse hyperalgesia, observed 4 h after carrageenan injection [71]. Another research group also investigated the anti-inflammatory effects of *S. officinalis* extracts in animal models. Their findings showed that sage extracts could reduce acute inflammation in a dose-dependent manner by inhibiting the synthesis or production of inflammatory mediators. However, they noted that the extent of this reduction did not match the efficacy of indomethacin, a nonselective COX inhibitor used as a reference drug, despite the fact that the doses of aqueous extracts were much higher than that of indomethacin [72]. Our study provides evidence that sage-based formulations may effectively alleviate inflammation and support the management of inflammatory skin conditions. These findings are consistent with the hypothesis that SNEDDSs can enhance the anti-inflammatory effects of active ingredients by improving their skin penetration and bioavailability. For example, it was also found in earlier studies that self-emulsifying systems, particularly those with a nanometer-sized droplet, showed superior anti-inflammatory effects when compared to conventional formulations in rats with induced paw edema [73,74]. In our previous study, it was also showed that SNEDDS-based formulations of natural anti-inflammatory agents, such as curcumin, demonstrated comparable anti-inflammatory effects to traditional drugs in in vivo models. This study emphasized that SNEDDSs could serve as a promising platform for enhancing the anti-inflammatory efficacy of topical treatments by improving the solubility and permeability of active compounds [44].

Our findings highlighted the versatility of SNEDDSs as a platform for enhancing the solubility, stability, and bioavailability of lipophilic drugs. The combination of SNEDDSs with natural extracts, such as *S. officinalis*, offers a promising avenue for developing multifunctional topical formulations with antioxidant, anti-inflammatory, and skin-nurturing properties. The promising results of this study open several avenues for future research. First, further in vivo studies on the long-term efficacy and safe formulations are necessary to confirm their potential for clinical applications. Additionally, studies focusing on the mechanistic pathways of how SNEDDSs enhance the anti-inflammatory and antioxidant effects of sage extract would provide valuable insights into the molecular actions involved, which could guide the development of more targeted therapies.

## 5. Conclusions

The results of the present study provide valuable insights into the formulation and optimization of topically applied systems for enhanced drug delivery. In summary, this study highlights the potential of self-emulsifying drug delivery systems, particularly those incorporating *S. officinalis* extract, for the development of topical formulations with improved bioavailability, anti-inflammatory, and antioxidant properties. The results provide a comprehensive understanding of how formulation parameters, such as surfactant content, pH, and texture, influence the performance of topical drug delivery systems. Moving forward, the combination of SNEDDSs with other active compounds may offer new possibilities for the treatment of a variety of dermatological conditions.

## Figures and Tables

**Table 1:** The composition of the formulated SNEDDS.

Compositions	Labrasol	Labrafil M	Transcutol HP	IPM	*S. officinalis* Extract
I_0_	30 g	-	30 g	25 g	-
I	30 g	-	30 g	25 g	15 g
II_0_	25 g	-	25 g	35 g	-
II	25 g	-	25 g	35 g	15 g
III_0_	-	30 g	30 g	25 g	-
III	-	30 g	30 g	25 g	15 g
IV_0_	-	25 g	25 g	35 g	-
IV	-	25 g	25 g	35 g	15 g

IPM: Isopropyl myristate.

**Table 2:** The compositions of creams containing *S. officinalis* with and without an SNEDDS.

Cream Ingredients	Salvia Cream	Cream + SNEDDS
Cremophor RH 40	2	2
Cetyl stearyl alcohol	5	5
Stearic acid	10	10
Propylene glycol	5	5
IPM	2.5	-
Preservative solution	1	1
Glycerin	5	5
SNEDDS III	-	10
*S. officinalis* extract	1.5	-
Distilled water	63.9	53.9

IPM: Isopropyl myristate.

**Table 3:** The compositions of gels containing *Salvia officinalis* with and without an SNEDDS.

Gel Ingredients	Salvia Gel	Gel + SNEDDS
Carbopol 974P	0.8 g	0.8
Triethanolamine	1 g	1
SNEEDS III	-	10
*S. officinalis* extract	1.5 g	-
Distilled water	96.7 g	88.2 g

**Table 4:** Particle size, PDI, zeta potential, EE%, and carnosol content of the formulated self-emulsifying systems. Values are expressed as mean ± S.D., *n* = 6.

Composition	Droplet Size (nm)	PDI	Zeta Potential (mV)	EE%	Carnosol Content (µg/mg)
I_0_	54.22 ± 2.33	0.201 ± 0.018	−26.32 ± 0.61		
I	64.92 ± 2.68	0.217 ± 0.018	−25.95 ± 0.42	93.12 ± 1.27	51.21 ± 0.69
II_0_	272.36 ± 5.02	0.198 ± 0.011	−17.33 ± 0.25		
II	291.10 ± 5.24	0.199 ± 0.012	−17.03 ± 0.36	82.66 ± 1.86	45.46 ± 1.02
III_0_	51.14 ± 2.74	0.169 ± 0.008	−29.54 ± 0.48		
III	62.26 ± 2.98	0.172 ± 0.010	−29.38 ± 0.26	95.73 ± 1.51	52.65 ± 0.83
IV_0_	304.86 ± 6.34	0.182 ± 0.012	−15.81 ± 0.67		
IV	318.03 ± 5.53	0.180 ± 0.011	−15.07 ± 0.29	85.03 ± 1.33	46.76 ± 0.73

PDI: Polydispersity index, EE%: Encapsulation efficacy (%).

**Table 5:** Particle size, PDI, zeta potential, EE%, and carnosol content of the formulated self-emulsifying systems 30 days after formulation. Values are expressed as mean ± S.D., *n* = 6.

Composition	Droplet Size (nm)	PDI	Zeta Potential (mV)	EE%	Carnosol Content (µg/mg)
I_0_	55.03 ± 1.86	0.202 ± 0.016	−25.79 ± 0.58		
I	65.21 ± 2.02	0.218 ± 0.020	−25.88 ± 0.52	93.04 ± 1.32	51.06 ± 1.23
II_0_	272.11 ± 4.88	0.200 ± 0.014	−17.02 ± 0.32		
II	293.26 ± 5.86	0.201 ± 0.014	−17.10 ± 0.34	82.62 ± 1.34	45.42 ± 1.21
III_0_	51.98 ± 2.02	0.172 ± 0.012	−29.04 ± 0.56		
III	60.96 ± 3.12	0.174 ± 0.014	−28.98 ± 0.52	95.66 ± 1.40	52.54 ± 1.16
IV_0_	307.34 ± 5.66	0.183 ± 0.012	−14.22 ± 0.56		
IV	319.24 ± 5.02	0.182 ± 0.012	−14.20 ± 0.48	84.98 ± 1.44	46.17 ± 1.33

PDI: Polydispersity index, EE%: Encapsulation efficacy (%).

**Table 6:** Particle size, PDI, zeta potential, EE%, and carnosol content of the formulated self-emulsifying systems 90 days after formulation. Values are expressed as mean ± S.D., *n* = 6.

Composition	Droplet Size (nm)	PDI	Zeta Potential (mV)	EE%	Carnosol Content (µg/mg)
I_0_	55.11 ± 2.01	0.202 ± 0.020	−25.31 ± 0.55		
I	65.66 ± 2.22	0.219 ± 0.020	−25.12 ± 0.56	93.03 ± 1.45	51.02 ± 0.98
II_0_	278.48 ± 5.14	0.205 ± 0.024	−16.54 ± 0.46		
II	298.08 ± 6.44	0.204 ± 0.022	−16.78 ± 0.40	82.60 ± 1.66	45.38 ± 1.33
III_0_	52.12 ± 3.14	0.172 ± 0.018	−29.01 ± 0.43		
III	62.33 ± 3.66	0.175 ± 0.014	−28.56 ± 0.55	95.63 ± 1.91	52.52 ± 1.44
IV_0_	313.03 ± 6.03	0.183 ± 0.022	−14.98 ± 0.56		
IV	320.12 ± 6.76	0.183 ± 0.020	−14.12 ± 0.42	84.93 ± 1.88	46.12 ± 0.98

PDI: Polydispersity index, EE%: Encapsulation efficacy (%).

**Table 7:** Results of the thermodynamic stability tests after six heating–cooling cycles (4 °C to 40 °C), three freeze–thaw cycles (−21 °C to 40 °C), and centrifugation at 2000 rpm for 30 min.

Composition	Heating–Cooling Cycles	Centrifugation	Freeze–Thaw Cycles
I_0_	√	√	√
I	√	√	√
II_0_	phase separation detected		
II	phase separation detected		
III_0_	√	√	√
III	√	√	√
IV_0_	phase separation detected		
IV	phase separation detected		

√—no visual change occurred.

**Table 8:** Droplet size and PDI values of the formulations after thermodynamic stability test. Values are expressed as mean ± S.D., *n* = 6. Statistical analysis was conducted using ordinary one-way ANOVA and Tukey’s multiple comparison test to compare formulations before and after thermodynamic stability test. Significant differences are indicated with * (*p* < 0.05).

Composition	Droplet Size (nm)	PDI
I_0_	54.91 ± 2.12	0.200 ± 0.016
I	65.06 ± 3.24	0.219 ± 0.020
II_0_	596.48 ± 13.14 *	0.769 ± 0.084 *
II	598.22 ± 18.53 *	0.874 ± 0.072 *
III_0_	52.03 ± 2.79	0.178 ± 0.017
III	62.30 ± 2.16	0.176 ± 0.016
IV_0_	503.76 ± 16.03 *	0.693 ± 0.066 *
IV	531.28 ± 21.07 *	0.708 ± 0.072 *

PDI: Polydispersity index.

**Table 9:** The pH values of *S. officinalis*–containing creams and gels immediately after formulation and after 30 and 60 days of storage at 21 °C. Values represent mean ± standard deviation (S.D.), *n* = 3.

Compositions	pH Value ± SD		
	Day 0	Day 30	Day 60
Salvia cream	5.75 ± 0.03	5.77 ± 0.02	5.78 ± 0.04
Cream + SNEDDS	5.92 ± 0.02	5.95 ± 0.04	5.95 ± 0.04
Salvia gel	6.58 ± 0.03	6.59 ± 0.03	6.58 ± 0.05
Gel + SNEDDS	6.32 ± 0.05	6.33 ± 0.05	6.35 ± 0.04

**Table 10:** Release rate and diffusion coefficient of carnosol in different compositions. Data are presented as mean ± S.D. (*n* = 6, *p* < 0.05). Statistical analysis was conducted using ordinary one-way ANOVA and Tukey’s multiple comparison test to compare formulations with and without SNEDDS. Significant differences are indicated with *.

Composition	Release Rate (k) (µg/cm^2^ × min½)	Diffusion Coefficient (D) (cm^2^/h)
Salvia cream	7.59 ± 0.30	0.04056 ± 0.00266
Cream + SNEDDS	17.94 ± 0.92 *	0.19448 ± 0.01834 *
Salvia gel	7.20 ± 0.57	0.01620 ± 0.00122
Gel + SNEDDS	8.42 ± 0.68	0.04208 ± 0.00143

**Table 11:** Comparison of release and diffusion profiles of formulations with and without an SNEDDS using difference factor (*f*_1_) and the similarity factor (*f*_2_).

Pairwise Comparison	*f*_1_ *^a^*	*f*_2_ *^b^*
Sage cream vs. Cream + SNEDDS	56.83	27.65
Sage gel vs. Gel + SNEDDS	42.80	49.44

^a^ *f*_1_ values of the difference factor calculation; ^b^ *f*_2_ values of the similarity factor calculation.

**Table 12:** TNF-α and IL-1β levels (pg/mg) in rat tissue lysates after 24 h treatment. Results were expressed as means ± SD. Ordinary one-way ANOVA followed by Dunnett’s multiple comparison test was performed to compare the samples with the negative control. The * indicates statistically significant differences at *p* < 0.05.

Sample	TNF-α (pg/mg)	IL-1β (pg/mg)
Negative control	38.76 ± 2.86	66.46 ± 5.32
Positive control	16.83 ± 2.08 *	47.59 ± 4.16 *
Cream + SNEDDS	22.80 ± 3.33 *	59.88 ± 5.93

Negative control: cream without *S. officinalis* extract (base cream); Positive control: methylprednisolone aceponate cream (1 *w*/*w*%).

## Data Availability

The data that support the findings of this study are available from the corresponding author (jozsa.liza@euipar.unideb.hu) with the permission of the head of the department, upon reasonable request.
